# The value of a redesigned clinical course during COVID-19 pandemic: an explorative convergent mixed-methods study

**DOI:** 10.1186/s12912-022-00872-8

**Published:** 2022-04-24

**Authors:** H. Ösp Egilsdottir, Lena Günterberg Heyn, Espen Andreas Brembo, Kirsten Røland Byermoen, Anne Moen, Hilde Eide

**Affiliations:** 1 Centre for Health and Technology, Faculty of Health and Social Sciences, University of South-Eastern Norway, Grønland 58, 3045 Drammen, Norway; 2 Institute of Health and Society, Faculty of Medicine, University of Oslo, Forskningsveien 2B, 0371 Oslo, Norway

**Keywords:** Nursing, Student nursing, Education, clinical, Computer simulation, Clinical competence, Nursing skills, Education nursing, Clinical reasoning

## Abstract

**Background:**

The COVID-19 lockdown in March 2020 had a significant consequence for nursing students worldwide including limited access to learning situations in clinical rotation. Therefore, this study aims to explore how an innovative redesign of a clinical course in a time of pandemic supported nursing students in learning the fundamentals of care in their first year. The redesign involved the transformation of a traditional hands-on clinical course into a technology-enhanced learning environment.

**Design:**

This was an explorative convergent mixed-methods study using both quantitative and qualitative methods.

**Methods:**

Twenty-four first-year nursing students responded to an online questionnaire with open-ended questions. Two nursing students and one faculty member participated in individual online interviews, and three faculty members participated in an online focus group interview. All the data were collected in June 2020. The quantitative data were analyzed using descriptive statistics and the qualitative data using content analysis. The GRAMMS guideline was applied.

**Results:**

The students achieved the learning outcomes regarding fundamental care, basic physical assessment skills, and clinical reasoning with the help of academic assignments, multimedia learning resources, and virtual patients. Further, six central aspects of the facilitator role in the virtual simulation were identified. The aspect that was considered most valuable involved uncovering the “red thread” between different areas of knowledge in the first year of nursing education; this supported the students to better understand how to think and talk like a nurse.

**Conclusion:**

This study offers insight into how a technology-enhanced clinical course can foster the learning of fundamental nursing care, basic physical assessment skills, and clinical reasoning skills; enhancing students’ preparedness for clinical hours. Virtual patients’ scenarios contributed to integrating different types of knowledge and skills that are important when providing nursing care for patients in clinical practice. This study also highlighted a gap in pedagogical competence among faculty members with regards to facilitating learning in a technology-enhanced learning environment. Study findings suggest promising pedagogical strategies that should be further developed post-pandemic, in response to the call for a renewal of nursing education using more technologically supported learning designs.

**Supplementary Information:**

The online version contains supplementary material available at 10.1186/s12912-022-00872-8.

## Background

In the spring of 2020, the COVID-19 pandemic resulted in global service disruptions, including lockdowns, shifting teaching, and learning to online platforms and a technology-enhanced approach [1, 2]. As many nursing curriculums mandate that 50% of student education takes place in clinical rotation [3], the pandemic had significant consequences for nursing students around the world. While some learning activities (e.g., lectures, discussions groups, and supervisions) were easily facilitated through online platforms, other learning activities (e.g., simulation and clinical skills training) were not well-suited to online facilitation. Access to learning situations in the clinical rotation was also very limited.

Given the above, and inspired by the Technological, Pedagogical, and Content Knowledge (TPACK) framework [4], we redesigned a first-year clinical course into a technology-enhanced learning environment focused on fundamental care, such as health assessment, clinical skills, and nursing interventions. A suite of mobile learning (mLearning) tools previously reported on in an earlier study [5] was introduced as the main content in the redesigned clinical course (RCC) to replace real-life patient encounters and clinical experiences with e.g. virtual patients and instructional videos. The current paper describes the RCC in detail and the students’ and faculty members’ experiences of participating in the course.

### Fundamental care and basic physical assessment skills (B-PAS)

The clinical rotation represents an important learning environment for nursing students to practice fundamental care and clinical skills [6, 7]. Feo et al. [8] have defined what constitutes fundamental care:

Fundamental care involves actions on the part of the nurse that respect and focus on a person’s essential needs to ensure their physical and psychosocial wellbeing. These needs are met by developing a positive and trusting relationship with the person being cared for as well as their family/careers (p.2295)

Kitson et al. [6] question whether the nursing profession has lost sight of how to value and view caring as a fundamental aspect of nursing, with the increased focus in modern nursing practice on cost-effectiveness, task orientation, and outcomes of care rather than how the care itself is provided. The authors offer a new vision for professional nursing practice and highlight the importance of fundamental care by introducing a conceptual framework: Fundamentals of Care (FoC). The FoC framework revolves around the nurse, patient, family, and context or health system in a multidimensional way [6]. Moreover, it focuses on how the nurse and the patient collaborate through a meaningful therapeutic relationship towards assessment, planning, implementing, and evaluating care related to fundamental care needs [6].

Health assessment with the integration of a wide range of physical assessment skills is an acknowledged core dimension of fundamental care [9]. The process of health assessment is closely linked to cognitive skills in nursing, including clinical reasoning and decision-making. The use of cognitive skills is grounded in pillars of professional knowledge, such as human bioscience knowledge (anatomy, physiology, pathophysiology, and pharmacology), fundamental nursing care, ethics, professional communication, and nursing documentation. However, newly graduated nurses lack confidence and competence in using all the physical assessment skills learned throughout their education, due to lack of role clarity [9] and lack of support and guidance from preceptors in clinical rotations [10, 11]. This may increase nurses’ resistance to using this core component of health assessment, which may reduce the likelihood that fundamental care needs being identified and met with appropriate nursing interventions [12].

Ideally, the range of physical assessment skills should be limited to what are considered the basic elements of physical assessment skills performed by a nurse, regardless of context [11, 13]. Therefore, a selection of physical assessment skills was chosen to be included in the basic physical assessment skills (B-PAS) curriculum [11]. Further, to provide a digital support for nursing students practicing B-PAS in clinical rotation, a Suite of mLearning tools were co-designed with nursing students from different educational years [5].

### Learning from virtual patient encounters and virtual simulation

A recent comprehensive systematic review identified the use and effect of virtual patients on student learning in health care educations [14]. Findings indicate that virtual patients are at least as effective as traditional education learning activities for knowledge outcomes, while the use of virtual patients seems to be more effective for skills outcomes [14].

Virtual patients are mediated through standardized computer software programs; students interact with the software by e.g. choosing different actions to communicate with the virtual patient, map the clinical situation, initiate fundamental care actions, administer medications, and consult a physician [14, 15]. The literature outlines a variety of methods for exploring students’ learning experiences related to using multimedia learning material and virtual patients, as well as methods for measuring learning outcomes. These learning outcomes mainly center around clinical reasoning skills, clinical decision-making, critical thinking, knowledge retention, communication, clinical skills performance, and students’ perceived confidence. Among the learning experiences explored in the literature are students’ satisfaction, attitudes, and technology-related concerns [14, 15].

Kononowicz et al. [14] point out that virtual patients can have a greater impact on knowledge outcomes when combined with the application of skills, particularly in problem-solving situations. Furthermore, virtual patients may also be a modality for learning clinical reasoning and critical thinking prior to clinical rotations, in order to prepare students for clinical hours [14]. Foronda et al. [15] reviewed the literature when mapping the use of virtual simulation (which includes virtual patients) in nursing education and found that this type of simulation improved student learning outcomes (knowledge, skills, satisfaction, critical thinking, and self-confidence) in 86% of the included studies (*N* = 80). More time spent on virtual simulation (virtual patients) correlated with increased benefits for learning. However, there was limited information concerning the time needed to achieve the described learning outcomes. Moreover, the feasibility and strength of the virtual patient scenarios appears rooted in the opportunity to repeatedly train clinical skills and clinical reasoning by re-visiting the same virtual patient scenario and re-assessing the clinical situation [16, 17]; in doing so, the students also continue refining their communication and history-taking skills [17].

The use of virtual patients in health care education can help standardize learning situations in clinical courses: this is beneficial both for ensuring that students have similar clinical learning experiences and thus preparedness for patient care, and helping them make up for lost clinical hours [14, 17, 18]. Georg and Zary [19] conceptualized how virtual patients can increase nursing students’ preparedness for clinical practice and clinical reasoning skills. In their work, the core aspects of the nursing role and fundamental care are mirrored in the utility of virtual patients in educational practice. Georg and Zary [19] also show how the virtual patient can be integrated into course design to highlight clinical reasoning skills based on feedback from faculty members, to identify the level of students’ knowledge and knowledge gaps. Further, Deschênes et al. [20] highlight how technology advancements can inform education strategies for learning clinical reasoning skills. In their findings, providing students with feedback based on a Socratic approach to questioning and modeling clinical reasoning skills supports students to understand what is involved in these cognitive processes and how to apply them in real patient encounters [20].

### Pedagogical perspectives to support the development of technology-enhanced courses

The TPACK framework offers a structure and context that describes the relationship and interaction between the three domains of technology, pedagogy, and educational content [4]. Every course design is a unique process, in which the interactions between these three domains will unfold differently [4]. Using a pedagogical framework to inform and support the integration of technology and pedagogy can help both faculty members and students to understand the purpose, aim, and learning outcomes in this kind of course design [7]. Increased use of technology-enhanced pedagogy in higher education indicates a transition from a teacher-centered approach towards a more student-centered and interactive approach [21, 22]. Six key factors are important to keep in mind when designing technology-enhanced courses: 1) the didactical competency for the design of suitable learning material to secure alignment with learning outcomes; 2) scaffolding student workload; 3) facilitating asynchronous learning processes; 4) student–faculty communication; 5) student–student communication, and 6) organizing support for students in how to use the different technical components in a technology-enhanced learning environment [2, 21].

Technology-enhanced learning material used in clinical rotation can support the learning of clinical skills and increase knowledge levels and students’ perceived confidence [7, 23]. Chuang et al. [23] found that using an instructional video delivered through a smartphone to support a specific clinical skill had a significant effect on students’ knowledge and performance of the skill compared to the control group (which was not given the instructional video). Furthermore, Stone et al. [7] reviewed the effect of podcast and multimedia materials on students’ level of knowledge, skills performance, satisfaction, and confidence: multimedia learning material seemed to have a greater impact on these learning outcomes for students in the lower grade range than those with higher grades.

Our review of the existing research revealed studies using multimedia learning material or virtual patients, or virtual simulation aimed for clinical learning. No studies were found that combined these learning activities as a substitution for a traditional clinical rotation or as a complementary learning activity in a clinical course. More evidence is therefore needed that provides insight into how these technological components can be combined to support students’ learning and utilizing of professional knowledge, clinical reasoning, and clinical skills, such as B-PAS.

## Methods

### Aims

This study aimed to explore how an RCC supported nursing students in learning fundamental care in their first educational year. Four research questions were formulated:

1. How much time did the nursing students spend using the available learning activities?
2. What was the nursing students’ self-reported confidence related to the B-PAS in the RCC?
3. What characterized the learning experiences in virtual patient encounters?
4. Which learning experiences did the nursing students and faculty members perceive as most prominent in the RCC?

### Design

**Fig. 1:**
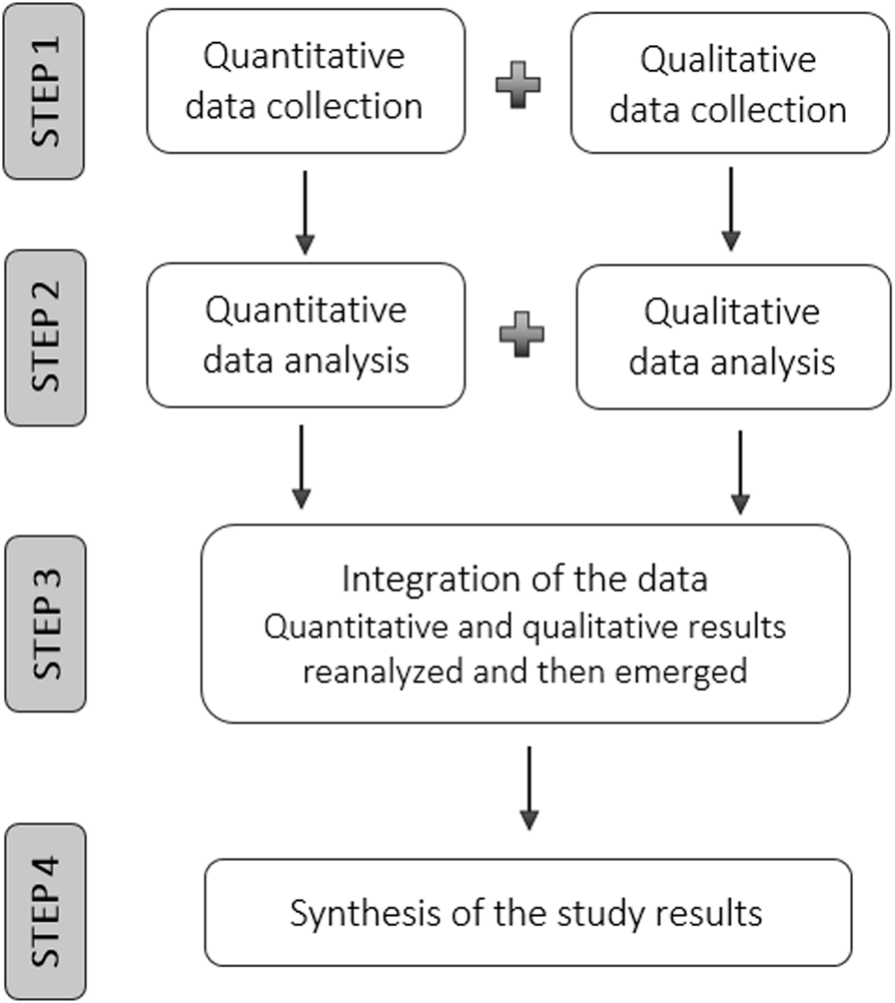
The explorative convergent mixed-methods design used in the study. Figure adapted from Creswell and Plano Clark (2018), p. 70

An explorative, convergent mixed-methods study was designed, using a questionnaire and interviews (individual and focus group) to collect data from students and faculty members (Fig. 1). A convergent design aims to collect different independent and complementary data about a specific research problem [24]. Hence, the data collection processes are not dependent on each other but rather carried out concurrently, as in this study. Creswell and Plano Clarke [24] argue that convergent design strengthen a research project by exploring the aim and the research questions from different perspectives. This was considered essential in this study, and was achieved by using two complementary research methods and exploring the study aim from the perspective of both students and faculty members.

### The redesigned clinical course

**Fig. 2:**
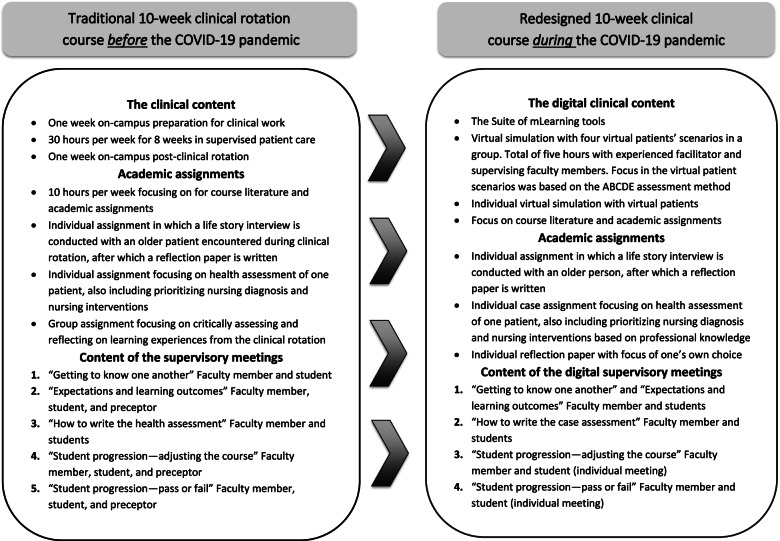
The clinical course before and after the redesign process

The point of departure was the curriculum for a 10-week clinical rotation course with specific learning objectives targeting aspects of fundamental nursing care, such as communication skills, learning systematic health assessment, and person-centred care for older patients and their families [25]. The content of the clinical course before the redesign is shown on the left side, in Fig. 2.

We began the redesign by assessing how the students could reach learning objectives in a technology-enhanced learning environment, by combining a Suite of mLearning tools [5] with traditional academic assignments (for an overview of the course content, see Fig. 2, right side). The following areas were prioritized: 1) taking the patient history, 2) learning B-PAS, 3) the health assessment, 4) communication skills, 5) fundamental nursing care and interventions, 6) human bioscience knowledge, and 7) clinical reasoning skills.

The RCC also included multiple digital interactions between the supervising faculty members and the nursing students. A commercial video conferencing system (CVCS) was used as a digital platform. The activities mediated through the CVCS involved different foci for supervision, student progress assessment, feedback on academic assignments, general student support, and the simulation with the virtual patient scenarios. The encounters in the CVCS were also an opportunity for faculty members to help students navigate the different learning activities, and to encourage them to use all available digital learning resources scaffolded through the 10-week RCC.

**Fig. 3:**
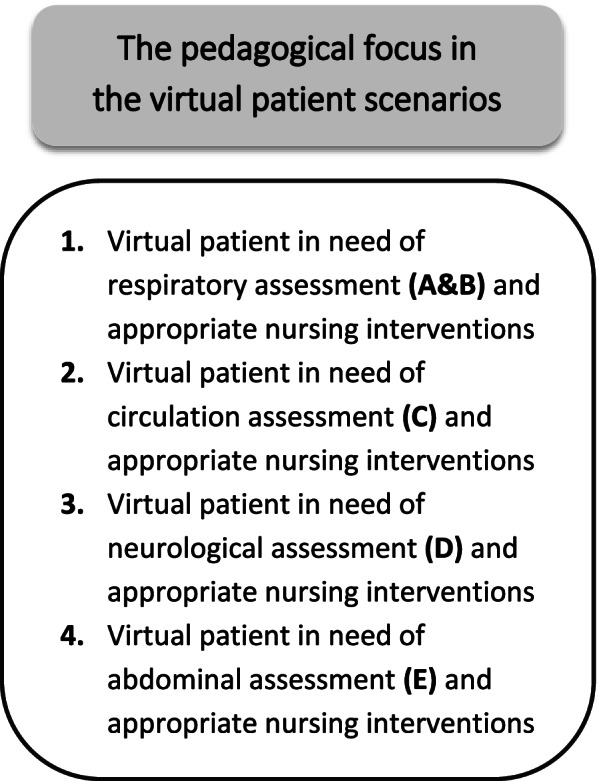
The virtual patient encounters

The simulation with virtual patients was conducted four times throughout the 10 weeks (Fig. 3). The selection of the virtual patient scenarios was based on the primary assessment approach: Airways (A), Breathing (B), Circulation (C), Disability (D), and Exposure (E) (ABCDE).

The four virtual simulation sessions were organized as shown in Table 1.

**Table 1:** Example of the structure of the virtual simulation sessions

Structure of the virtual simulation sessions			
Pedagogical focus	Pre-assignments	Virtual simulation sessions	Learning outcomes
Virtual patient in need of respiratory assessment (A&B) and appropriate nursing interventions	Three virtual patient cases available for as many encounters as the students wanted. One of these cases was marked as the selected case for the group simulation session	75 min group simulation with 8–12 students. Students were divided into active participants and observers throughout the session. • 5–10 min intro and “small talk” • 45–50 min virtual patient encounter and in-depth debriefing • 5–10 min “finishing up” and “how did it go?”	Identify the patient’s resources, basic needs, and health condition by using a systematic approach and health assessment. Perform basic physical assessment with focus on respiratory assessment. Verbalize knowledge on characteristics of diseases related to elderly patients

The same experienced facilitator was responsible for all four sessions and had access to the virtual simulation program via computer (Table 1). By sharing the screen, all the participants could see and hear everything happening in the virtual patient scenario. In addition, the faculty members responsible for supervising the nursing students also participated in the virtual simulation sessions. Their role was to contribute to the ongoing discussions and give advice to the students in their encounters with the virtual patient. This involvement gave the faculty members a unique opportunity to follow up on specific elements later in the supervisory meetings with students. The facilitator executed all the actions throughout the simulation. The students who had an active role in the simulation session decided together which actions to take. The facilitator paused the simulation whenever it was pertinent to explore relevant professional knowledge underpinning the actions and clinical reasoning, by using interrogative words like “why,” “how” and “what.” The students were continuously encouraged and stimulated to verbalize their knowledge, reflections, and ideas throughout the session with the facilitator, participating faculty member, and fellow students. The students were also advised to use any types of aids, course literature, online resources, or active reflections with peers.

In addition, the RCC included mandatory academic assignments (Fig. 2, right side). The aim of the assignments was the same as in the traditional clinical rotation course, with a few adjustments. A life story interview was conducted via telephone instead of face-to-face. The nursing students could choose whom to interview, as long as it was an older person. All the students received the same patient case as a starting point for the case assignment; further, they each submitted the assignments individually and were rated pass/fail by the supervising faculty member. The researchers involved in the study had no role in the evaluation or rating of the students’ assignments.

The redesign process also included exploration of what could constitute as a patient encounter in a technology-enhanced clinical course. This process led us to suggest that the following elements could be viewed as patient encounter: the virtual patients, the case assignment, the life story interview, instructional videos and a selection of assignments in the Massive Open Online Course (MOOC) which were available in the Suite of mLearning tools [5].

### Participants

The participants in the study were first-year nursing students enrolled in a three-year bachelor program and program faculty members, all at the same university in Norway. All nursing students registered in the RCC (*N* = 55) were invited to participate in both the quantitative and qualitative parts of the study. The students received written and oral information (via a video presentation) about the study through the university’s learning management system (LMS). Upon completion of the 10-week RCC, the students were invited to answer the questionnaire and to participate in the interviews.

Twenty-four nursing students agreed to participate and answered the study-specific questionnaire (41.8% response rate). All but one was female. Thirteen students provided textual feedback about the strengths and weaknesses of the RCC, and two female students participated in the online individual interviews. The majority of the students (54.2%) were younger than 24 years of age (Table 2).

**Table 2:** Overview of the age range for the nursing students who answered the questionnaire

Age range	Total (%)
20–24	13 (54.2)
25–29	3 (12.5)
30–34	2 (8.3)
35–39	1 (4.2)
40–44	2 (8.3)
45–49	1 (4.2)
50–54	1 (4.2)
Missing	1 (4.2)

At the end of the RCC, all faculty members who were responsible for the supervision of the students in the RCC (*N* = 16) received oral (via CVCS) and written information about the study and an invitation to participate in the qualitative interviews. Three faculty members participated in the online focus group interview and one was interviewed individually online. All the faculty members were females, ranging in age from 47 to 60 years.

### Data collection

All the data were collected at the beginning of June 2020, just after the completion of the 10-week RCC. The student questionnaire, which also included open-ended questions, was administered concurrently with the qualitative interviews with students and faculty.

#### The student questionnaire

For this study, a 62-item questionnaire divided across 11 sections was developed. The questionnaire was not validated prior to its use. In section 2, the students were asked to estimate the time spent on each learning activity per week in the RCC. For items in sections 3 to 10 (see Tables 5, 6, 7, 8 and 9), a 5-point Likert scale was applied with the following options: “disagree,” “disagree a little,” “not sure,” “slightly agree,” and “agree.” Section 11 contained three open-ended questions, where the students were asked to describe what they perceived as strengths and/or weaknesses of the RCC, and note any other comments related to the course. These descriptions were included in the qualitative data material.

#### The interviews and open-ended questions

**Fig. 4:**
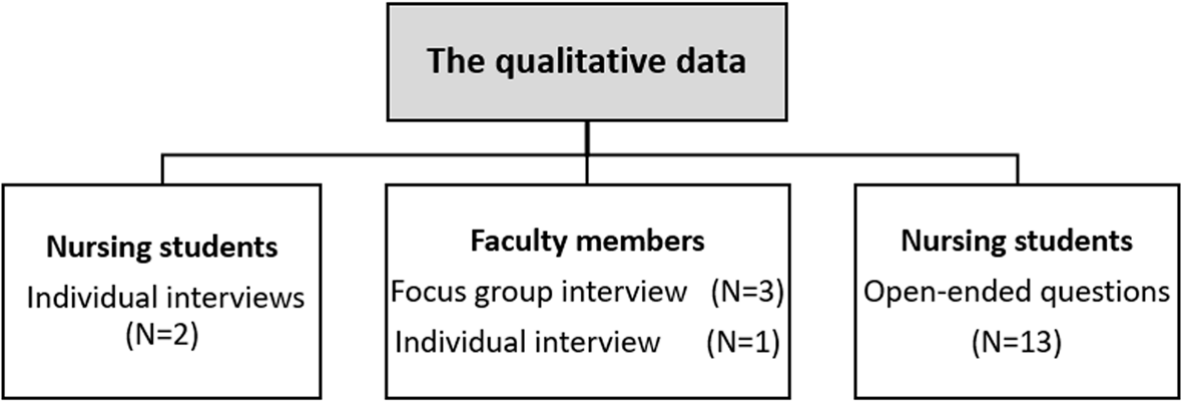
The extent of the qualitative data

All the interviews were conducted in the CVCS to comply with COVID-19 restrictions. The interview guide was structured thematically and the follow-up questions under each theme sought to further explore the participants’ experiences related to the rapid shift from traditional clinical rotation course to an RCC, their own digital competence, perceived strengths and/or weaknesses of the RCC, experiences with the virtual simulation and the virtual patients, and supervision of students over the 10 weeks in the RCC. Figure 4 gives an overview of the qualitative data.

### Ethical considerations

The Norwegian Centre for Research Data (NSD) approved the study (Ref. nr. 674624). All the participants received both written and oral information about the study. The content of the information contained a) aim of the study, b) how to participate, c) the voluntary aspects of the participation, d) the opportunity to withdraw at any time, and e) how the informed consent was collected. The information to the students also included information about returning the online questionnaire was an active form for consent for participation in the study. Due to the COVID-19 restrictions, the students and faculty members participating in the interviews received written information in advance of the interview including; a) information of the video recording, b) information that the video recording would be deleted when the research project had ended, and c) that the recordings from the interviews were stored in secure research domain at the university. The participants gave their consent before the time and date for the interviews were scheduled. All the participants were asked again before starting the video recording and were given the opportunity to reserve themselves from being recorded. However, all the participants consented verbally, as well as actively consenting by “clicking” on pop-up notification allowing the recording to start. Further, the researchers responsible for the study were not involved in any formal evaluation of students’ performance in the RCC; hence, students’ participation in the study had no impact on their pass/fail outcomes in that course.

### Data analysis

#### Analysis of the questionnaire data

The statistical software package SPSS 26 was used to perform the descriptive and frequency analyses. We calculated mean scores, standard deviation, and range for each item in the section 3 to 10 in the questionnaire.

#### Analysis of the interviews and open-ended questions

Qualitative inductive content analysis was used to analyze the qualitative data [26]. All the interviews were transcribed verbatim and read thoroughly to obtain an overview of the data. The overall process of analysis involved open coding, grouping codes into subcategories, and then interpreting the subcategories into categories (Table 3). These categories were then abstracted into the main categories.

**Table 3:** Example of the relationship between the questions in the interview guide and the main categories

Questions from the interview guide	Main categories	Faculty member quotes
What are the possibilities and limitations of simulation with virtual patients?	1. The importance of recognizing the students’ vulnerability 2. The responsibility of the facilitation in the virtual simulation sessions	*“The students made themselves vulnerable, and threw themselves into the unknown—and they mastered it! I really applaud them for that.”* “*I like that the focus is mainly on the process where one stops and dwells on ‘What does it mean when the inhalation or the exhalation has a stridor sound?’ or ‘What exactly is blood pressure and what role does blood pressure have in the circulation system?’ or ‘What does it mean when blood pressure drops?’ You get the opportunity to systematize your knowledge and puzzle it together, which I find very exciting in the immersive simulation.”*
What are the strengths or weaknesses in the redesigned clinical course?	3. The value of promoting students’ preparedness for future patient encounters	*“We have talked about how to palpate and to inspect—and it is obvious that they* (the students) *will not get the same impression as they would in real-life patient encounters and therefore lack the opportunity to perform the clinical skills ‘hands on.’ We cannot replace that even if we talk about it.”*
Thinking about your own digital literacy: Has it changed after the participation in the redesigned clinical course, and if so, how?	4. The need for more and new pedagogical competence	*“Participating was useful to gain insight about what the students really know when they participate in a simulation like this. I think that you get a good impression of the students’ performance through the virtual simulation.”*

#### Convergent mixed-methods analysis

In mixed-methods research, it is important to work towards an integration of the quantitative and qualitative data; as such, after analyzing the different data sets separately, we explored the results collectively to look for any congruence or discrepancy in the data sets, as suggested by Creswell and Plano Clark [24]. This second stage of data analysis revealed new understandings and represented an additional level of synthesis of the overall findings.

### Validity, reliability, and analytical rigor

The questionnaire was critically reviewed by the co-authors of this paper. All unclear items were discussed, and a consensus was reached regarding the formulation of each item. Two researchers (HÖE/LGH) read the interviews separately. The open coding, grouping the data into subcategories, and identifying categories were also done separately by HÖE and LGH. Then, the same two researchers completed the abstraction of the data into main categories collaboratively. All researchers finalized the convergent analysis and integrated the data according to the research questions.

## Results

In the following section, the results from the quantitative and qualitative analyses are presented separately to emphasize the data provided by the two data collection methods. Then, in line with convergent design [24], the coherence of the two data sets will be addressed.

### The quantitative results

#### Students’ use of the available learning activities

We asked the students to estimate the amount of time spent on the different learning activities during each of the 10 weeks. The data indicate variety in how many hours the students spent on these activities (Table 4). The most time was spent on academic assignments, followed by reading course literature. The students spent an average of 4.4 h per week on the material aimed at supporting the learning of B-PAS, and 3.5 h engaging with the virtual patients. The students spent the least amount of time on the MOOC and the podcasts.

**Table 4:** Overview of hours spent per week on available learning activities

Learning activity	N	Mean (SD) hours per week	Range hours per week
Academic assignments	21	14.9 (9.4)	3–45
Reading course literature	18	8.3 (6.1)	0–20
Video lectures and instructional videos	21	4.4 (2.7)	0–10
Simulation with virtual patients	19	3.5 (2.6)	1–10
MOOC	14	1.4 (1.8)	0–6
Podcasts	20	1.3 (1.2)	0–4

#### Nursing students’ perceived confidence related to learning B-PAS

Most of the students agreed that the multimedia learning material helped them gain a better understanding of the appropriate use of B-PAS in future patient encounters (Table 5).

**Table 5:** The importance of the multimedia learning material for understanding how to use B-PAS in future patients encounters

The multimedia learning material	N	Mean (SD)	Range
The video lectures helped me understand how I can use B-PAS in patient encounters	24	4.6 (0.58)	3–5
The instruction videos helped me understand how I can use B-PAS in patient encounters	24	4.6 (0.65)	3–5

Overall, the students agreed that participation in the RCC had increased their confidence in performing the different examination techniques and foci related to B-PAS (Table 6). The students perceived themselves as confident in inspecting and auscultating the thorax, inspecting and percussing the abdomen, assessing the motoric system, balance, and coordination of the patient, and assessing peripheral sensibility. However, the students perceived themselves as least confident in auscultating the heart sounds, percussing the thorax, and testing the cranial nerves.

**Table 6:** Nursing students’ perceived confidence in performing B-PAS in future patient encounters

The focus in the multimedia learning material		N	Mean (SD)	Range
**The respiratory system**	**I am confident that I can…**			
	inspect properly after viewing the multimedia learning material	23	4.3 (1.02)	1–5
	palpate properly after viewing the multimedia learning material	23	4.2 (0.10)	2–5
	percuss properly after viewing the multimedia learning material	24	4.0 (1.25)	1–5
	auscultate properly after viewing the multimedia learning material	24	4.3 (0.86)	2–5
**The circulation system and the heart**	**I am confident that I can…**			
	inspect properly after viewing the multimedia learning material	24	4.2 (0.93)	2–5
	palpate properly after viewing the multimedia learning material	24	4.1 (0.93)	2–5
	auscultate properly after viewing the multimedia learning material	24	4.0 (1.12)	1–5
**The abdominal system**	**I am confident that I can…**			
	inspect properly after viewing the multimedia learning material	24	4.3 (0.99)	2–5
	auscultate properly after viewing the multimedia learning material	24	4.2 (0.92)	2–5
	palpate properly after viewing the multimedia learning material	24	4.2 (0.85)	2–5
	percuss properly after viewing the multimedia learning material	24	4.3 (0.71)	3–5
**The neurological system**	**I am confident that I can…**			
	test the cranial nerves properly after viewing the multimedia learning material	24	4.0 (1.23)	1–5
	test the motoric system properly after viewing the multimedia learning material	24	4.4 (0.82)	2–5
	test balance and coordination properly after viewing the multimedia learning material	24	4.3 (0.82)	2–5
	test peripheral sensibility properly after viewing the multimedia learning material	23	4.3 (0.92)	2–5

#### The learning experiences from the virtual patient encounters

The questionnaire data show that the students experienced the virtual patient encounters as a meaningful learning activity (mean 4.6, SD 0.58, range 3–5) to support the development of confidence in fundamental nursing care (Table 7). Further, the students reported the virtual simulations sessions as a safe learning environment (mean 4.5, SD 0.8, range 2–5), and that it was useful to have the faculty members participate, as well (mean 4.8, SD 0.51, range 3–5). In particular, two items stood out with the highest mean score and one item with the lowest. Despite a wide range of students’ scores on some of the items, the majority scored “slightly agree” or “agree” on all items related to the virtual patient encounters.

**Table 7:** Nursing students’ perceived confidence and mastery of fundamental care in the virtual patient encounters

The simulation with virtual patients helped me…	N	Mean (SD)	Range
become more confident in how to systematically map the patient clinical situation	24	4.5 (0.83)	2–5
understand when to use B-PAS in future patient encounters	24	4.6 (0.71)	2–5
become more confident in how to collect subjective data in patient encounters	24	4.1 (1.35)	1–5
become more confident in how to assess subjective data in patient encounters	24	4.3 (0.10)	1–5
become more confident in how to collect objective data in patient encounters	23	4.6 (0.90)	1–5
become more confident in how to assess objective data in patient encounters	24	4.5 (0.59)	3–5
become more confident in how to assess collected data and to reason possible cause(s) for the patient’s clinical situation	23	4.4 (0.94)	1–5
become more confident in clinical decision-making	23	4.2 (1.09)	1–5
experience mastery in the way I verbalize professional knowledge in future patient encounters	24	4.2 (0.93)	2–5
become more confident about my knowledge in anatomy and physiology	24	4.4 (0.71)	3–5
become more confident about my knowledge in pathophysiology and pharmacology	24	4.2 (1.09)	1–5
develop clinical reasoning skills	23	4.5 (0.73)	3–5
develop clinical decision-making skills	24	4.5 (0.78)	2–5

The questionnaire data indicate that the nursing students had positive experiences with the academic assignments (Table 8). The case assignment stood out as the assignment that the students felt contributed most to their increased confidence in systematically mapping the patients’ clinical situation. In contrast, the reflection paper and life story interview were the assignments that received the lowest mean scores.

**Table 8:** The importance of the academic assignments on students’ learning in the RCC

The different academic assignments	N	Mean (SD)	Range
The case assignment helped me feel more confident in systematically mapping the patient clinical situation	24	4.8 (0.42)	4–5
The case assignment helped me feel more confident in knowing the difference between subjective and objective data	24	4.6 (0.89)	1–5
The case assignment helped me feel more confident about my knowledge in fundamental nursing	23	4.6 (0.59)	3–5
The case assignment helped me feel more confident about my knowledge in anatomy and physiology	22	4.4 (0.95)	1–5
The case assignment helped me feel more confident regarding my knowledge in pathophysiology and pharmacology	23	4.3 (1.06)	1–5
The reflection paper helped me become more conscious about own learning processes	21	4.1 (0.96)	3–5
The life story interview helped me become more confident in the communication with older people	22	4.1 (1.06)	1–5
The life story interview helped me become more confident about my knowledge in communication skills	23	4.3 (0.59)	1–5

The majority of the nursing students also reported (Table 9) that the overall content of the RCC gave them increased confidence regarding fundamental care and human bioscience knowledge, which are specific parts of professional knowledge.

**Table 9:** Nursing students’ perceived confidence related to fundamental care and human bioscience knowledge

The redesigned clinical course helped me…	N	Mean (SD)	Range
feel more confident about my knowledge in fundamental care	22	4.6 (0.59)	3–5
feel more confident about my knowledge in anatomy and physiology	23	4.1 (1.14)	1–5
feel more confident about my knowledge in pathophysiology and pharmacology	21	4.2 (0.93)	1–5

### The qualitative results

The results from the interviews with the nursing students are presented first, followed by the faculty members’ perspectives.

#### Students’ learning in the RCC

Four main categories were identified in the analysis of the interviews with the students, combined with the answers from the open-ended question in the questionnaire: 1) exploration of professional knowledge fosters development of clinical reasoning skills; 2) caring facilitation contributes to building confidence; 3) using the full potential of the virtual patients is important; and 4) a flexible teaching and learning approach strengthens professional knowledge.

##### Category 1: Exploration of professional knowledge fosters the development of clinical reasoning skills

The students highlighted the virtual simulation sessions as a safe learning environment for sharing their thoughts and reflections with the other participants. In their experience, sharing their reflections and knowledge, and revealing knowledge gaps, made them vulnerable in front of the other students, facilitator, and faculty members. As one of the students explained:

*The participation is stressful because you are worried about getting it* (the answers or actions) *wrong. You think that maybe you should avoid saying anything because everybody* (the other participants) *can think…well they might think something. But you cannot focus on that or let that intimidate you, just carry on and do it.*

However, at the same time, they expressed the importance of having the courage to be vulnerable in this way. One student noted:

*It’s not just a form of communication. This is a way that makes you more aware of your own clinical reasoning because you verbalize it* (professional knowledge)…*so this becomes a method of learning. I think it’s important to talk out loud. You can be in your head, in your brain, but you cannot get it* (professional knowledge) *out. You know how it* (the body) *works and you have a lot of knowledge embedded within you, but when it comes to talking about it—that is when it becomes challenging.*

The students felt that the virtual patient encounters were especially helpful for realizing the importance of taking note of clinical cues that appeared during the scenario—and then determining the importance of those cues, and the appropriate actions. One student said: *“In the virtual patient encounters, you can simulate acute situations, for example a stroke, and get your level of knowledge and performance confirmed.”* The students also valued the learning activities that were part of the preparation phase, like refreshing professional knowledge within anatomy, pathophysiology, pharmacology, and fundamental nursing interventions. As this student wrote: *“We achieved basic competence in B-PAS and we got the opportunity to link different subjects that we have learned and use it in a specific clinical situation.”* Further, the nursing students shared the view that engaging in virtual patient encounters helped them understand how a systematic approach in assessing the clinical situation can aid them in developing clinical reasoning skills.

##### Category 2: Caring facilitation contributes to building confidence

It was important for the students that the facilitator clearly expressed their expectations regarding the students’ contribution, participation, and communication. As one student related:

*The facilitator said, ‘Now we are going to work together—you are the ones who suggest what to do and then we are all going to talk together and reflect on the different actions in the case’. Then you just breathe and relax because you think, ‘We are going to do this together and we are all in the same boat.*

The students elaborated further on the importance of how the facilitator challenged the students with questions aimed at stimulating verbalization of knowledge via reflections in and on actions. These questions were asked in a kind and non-judgmental way, regardless of whether or not the students knew the answer. One of the students described this as follows:

*In the virtual patient encounters you could choose actions without doing serious harm to the patient and you can try out different actions—it was never like ‘No, this was the wrong answer!’ When the facilitator asked, and we* (the students) *replied, perhaps not with the exact right answer, the facilitator corrected us in a very caring way. It was reassuring to have the facilitator there.*

This also underlines how the students valued being corrected whenever they failed to detect and/or interpret clinical cues and to act accordingly.

##### Category 3: Using the full potential of the virtual patients is important

The students contributed valuable insight into how the virtual patients could be used to integrate theory and practice, and what the role of the faculty should be. From their perspective, a good pedagogical design with a well-structured plan for the virtual patient encounters was essential to optimize the learning experiences and outcomes in the RCC. The students envisioned more frequent use of virtual patients during the first year of the nursing program. After becoming familiarized with this learning activity in the RCC, they would prefer having access to the virtual patients as an additional learning resource in other courses. One student said:

*I would prefer having it* (the virtual patients) *more often and for a bit longer than 1 hour and 15 minutes. Perhaps for one and a half hours. That would allow us to talk in more detail about the assessment related to the inspection, palpation, auscultation, and percussion.*

Another student said:

*The virtual patients should be a regular thing within the different courses. Then you would have a specific case to work on, both on your own and with your study group.*

Furthermore, the students experienced the virtual patient as a learning activity that revives the focus on human bioscience knowledge. Thus, they recommended that the physiological changes or clinical cues in the virtual patient scenarios should be the main focus of the discussions and cognitive reasoning skills grounded in professional knowledge. As this student pointed out:

*I have had an extra focus on the pathophysiology part. I have read about all the diseases related to every case and it is motivating to try and see how the patient is doing and how you can try to fix the situation.*

The students agreed that it was helpful to have the faculty members responsible for supervision in the RCC also involved in the virtual simulation sessions. One of the students elaborated on this:

*It is great to meet more frequently and with faculty members. It is great when the faculty members came with inputs. They participate at the same level as you. You do not feel that you are on the same level, but you get more out of it* (the virtual patient encounter) *when the input or feedback comes from more than one nurse or faculty member—you learn more. I think that the faculty members are there to help and to reflect together with you.*

The involvement of the faculty members was generally experienced by the students as supportive; moreover, by sharing their professional knowledge, they added new perspectives in the virtual patient encounter.

##### Category 4: A flexible teaching and learning approach strengthens professional knowledge

The structure of the RCC gave the students well-appreciated flexibility in terms of time, space, and place for engaging in the learning activities; they felt it was tailored to their everyday life activities and obligations. The following were written in response to the open-ended questions:

*It was easier to keep up in the lectures, easier to take notes without being interrupted by other students. I could also sit anywhere, like outside and I felt more motivated to engage.*

and *“You can engage in the learning activities whenever it fits with your daily plans.”* However, the students were clear that a technology-enhanced clinical course that relies on a high level of interactivity demands self-discipline to “get the job done”: *“You have to have the self-discipline to do it* (the learning activities) *because it is important not to think that you will do it later but to actually do it now.”* The students found it valuable that the instructional videos and the MOOC involved real people, especially when the students were not meeting real patients in this clinical course. One student said: *“I think that it was cool that it was a real person. That made it a bit more real and more motivating to engage in the course.”* Another student said: *“It was so good to see how the examination techniques were done on a real person.”* However, the students missed having social interaction, as they were not physically present with their fellow students. This aspect was evident both in the feedback they gave in the interviews and the open-ended questions. As one student asserted: *“The social distance and no physical presence are difficult.”* And another explained,

*One of the strengths of the RCC was that the learning material is accessible all the time and you can revisit whenever it suits you, but I miss the social aspect of being a student.*

Overall, however, the students reported that they felt their level of professional knowledge was strengthened and would benefit future real-life patient encounters.

#### Faculties’ roles and responsibilities as learning facilitators in the RCC

The interviews with the faculty members revealed three main categories: 1) the responsibility for facilitating reflection to stimulate integration between professional knowledge and clinical skills; 2) to promote for students’ preparedness for future patient encounters; and 3) the need for new pedagogical and technological competence.

##### Category 1: The responsibility for facilitating reflection to stimulate integration between professional knowledge and clinical skills

The faculty members agreed that the facilitators’ main responsibility in the virtual patient encounters was to stimulate reflection on elements of professional knowledge, and how the integration with clinical skills contributed to the development of clinical reasoning skills. They further emphasized how the virtual patient encounters provided the students with unique opportunities to consciously and systematically “explore” the patient’s current physiological status, and in that process, also notice clinical cues when sudden changes occurred in the scenario. One of the faculty members said:

*It is a useful thing to do because it is so concrete. I believe that it gives them* (the students) *the opportunity to learn a systematic approach and I see in the other cases that you can learn clinical reasoning skills as well.*

Furthermore, the faculty members saw the value of being able to reflect ‘in and on actions’ and how that revealed the core function of the facilitator role in the virtual patient encounters. As this faculty member explained:

*I like that the focus is mainly on the process where one stops and dwells on ‘What does it mean when the inhalation or the exhalation has a stridor sound?’ or ‘What exactly is blood pressure and what role does blood pressure have in the circulation system?’ or ‘What does it mean when the blood pressure drops?’ You get the opportunity to systematize your knowledge and puzzle it together, which I find very exciting in the digital simulation.*

The faculty members were also aware that the students were exposed and vulnerable in the simulation sessions. One of the faculty members noted: “*The students made themselves vulnerable and jumped into the unknown—and they mastered it! I really applaud them for that*.” Moreover, faculty members noted their surprise that some of the first-year nursing students already showed an ability to use clinical reasoning skills in virtual patient encounters. Finally, they stressed the benefits of using virtual patients in education, and how the virtual patients can be used to link different aspects of the nursing role, like fundamental care, health assessment, communication skills, and clinical skills.

##### Category 2: Promoting students’ preparedness for future patient encounters

Given the circumstances surrounding COVID-19, the faculty members agreed that the RCC was an appropriate substitute allowing the nursing students to fulfill their study progression requirements. In their opinion, the overall organization and scaffolding of the learning activities would benefit the students in future real-life patient encounters. One faculty member said:

*I primarily think that the students have learned how to work systematically by using the tool* (the virtual simulation)*, which represents in a way a clinical context. I think that they* (the students) *will benefit greatly from this* (the virtual patient) *when they meet patients later on. I think this is a major strength of this specific learning activity.*

The faculty members also highlighted the shortcomings of being unable to participate in real-life clinical contexts, including normal verbal and non-verbal communication with patients and listening to their personal stories. Here, they felt the students also lacked the dimension of learning from environmental impressions, such as different smells, sounds, and being able to touch the patients physically. One faculty member said:

*We have talked about how to palpate and to inspect—and it is obvious that they* (the students) *will not get the same impression as they would in real-life patient encounters and therefore lack the opportunity to perform the clinical skills ‘hands-on’. We cannot replace that even if we talk about it.*

In other words, the faculty members felt that, though the RCC provided the students with meaningful learning activities, this type of clinical course could never replace real-life learning experiences in a clinical context.

##### Category 3: The need for new pedagogical and technological competence

The faculty members elaborated on the importance of having the pedagogical and digital competence required to redesign a clinical course strategically. They acknowledged the complexity of scaffolding different learning activities, planning for student supervision, supporting the use of different digital learning resources, and creating a safe learning environment that promotes discussion, reflection, and learning. They experienced it as challenging to support and motivate student engagement, to promote useful and meaningful communication, and to decide which methods are best-suited to assessing students’ performance and level of knowledge in an RCC. One faculty member said: *“I discovered and learned that you had to be pretty lively and entertaining to avoid having the communication, supervision or teaching become monotone.*” The faculty members valued participation in the virtual patient encounters because they experienced it as essential to get the right “feeling” and understanding of how knowledgeable the students were in the virtual patient encounters. One faculty member stated that:

*Participating was useful to gain insight into what the students really know when they participate in a simulation like this. I think that you get a good impression of the students’ performance through the virtual simulation*.

Further, the faculty members experienced it as important for their role to help the students work towards reaching the learning outcomes in the RCC and that the available learning activities in the RCC were an invaluable resource. A few of the faculty members were surprised by how inexperienced some students appeared to be, regarding seeing each other on-screen. One of them said:

*There are people in the group who have seen each other just in a sports top in the skill lab! It is a bit strange to notice what happens when we all of a sudden are seeing each other on a screen. Suddenly we cannot show ourselves and the screens turn black*.

The faculty members agreed that these “black screens” made them feel insecure, and emphasized that it is challenging to supervise students through black screens—hearing only a voice without a face.

### The integration of the mixed-methods results

The results from the two data sets were coherent and complementary in many ways (Table 10). Concerning the integration of the data, Creswell & Plano Clark [24] argue that coherence between quantitative and qualitative results can increase the credibility of the overall findings from a mixed-methods study. The quantitative data highlight how the students perceive their confidence related to understanding when it is appropriate to use B-PAS in future patient encounters, and how the virtual patient scenarios helped students learn structured patient assessment. Complementary findings emerged in the qualitative findings. The students and faculty members had similar experiences and perspectives regarding learning in the virtual patient encounters and about the RCC. It is worth highlighting that students’ clinical reasoning skills and learning of structured patient assessment were emphasized in both groups. In addition, both students and faculty members asserted that the RCC was a necessary substitutional “clinical” course during the pandemic. However, it was discussed that the art of nursing also needs to be learned in authentic hands-on patient encounters in real-life clinical practice. The only discrepancy between the data sets was that faculty members shared their reflections regarding their role and competence as educators and facilitators in a RCC.

**Table 10:** The coherence and discrepancy in the data sets

Quantitative results	Qualitative results	
Students’ perspectives	Students’ perspectives	Faculty members’ perspectives
Use of the available learning activities	**3)**^**a**^ Using the full potential of the virtual patients is important **4)** A flexible teaching and learning approach strengthens professional knowledge	**3)**^**b**^ The need for new pedagogical and technological competence
Perceived confidence in using B-PAS in future patient encounters	**2)** Caring facilitation contributes to building confidence **3)** Using the full potential of the virtual patients is important	**1)** The responsibility for facilitating reflection to stimulate integration between professional knowledge and clinical skills
Learning experiences from the virtual patient encounters	**1)** Exploration of professional knowledge fosters the development of clinical reasoning skills **2)** Caring facilitation contributes to building confidence **3)** Using the full potential of the virtual patients is important **4)** A flexible teaching and learning approach strengthens professional knowledge	**1)** The responsibility for facilitating reflection to stimulate integration between professional knowledge and clinical skills **2)** Promoting students’ preparedness for future patient encounters
Importance of the academic assignments in the RCC for students’ learning	**1)** Exploration of professional knowledge fosters the development of clinical reasoning skills	**3)** The need for new pedagogical and technological competence
		**3)** The need for new pedagogical and technological competence

^a^ The numbers indicate the main categories in the qualitative results regarding the students’ perspectives

^b^ The numbers indicate the main categories in the qualitative results regarding the faculty members’ perspectives

## Discussion

This study contributes to the literature with three main findings: 1) a technology-enhanced learning environment can enhance students’ preparedness for future clinical practice; 2) facilitation in virtual patient encounters can uncover the “red thread” within nursing education, thereby fostering early development of clinical reasoning skills; and 3) new technology-enhanced learning activities call for new pedagogical competence among faculty members.

### A technology-enhanced learning environment can enhance students’ preparedness for future clinical practice

The nursing students experienced that the different learning activities in the RCC contributed to learning fundamental care in nursing, despite being conducted in a technology-enhanced learning environment. The students highlighted the case assignment, the multimedia learning material, and the virtual patients being especially helpful. It was noted that although “hands-on” B-PAS training was not possible, another important dimension of learning B-PAS was achieved: the students acquired an understanding of *when* and *why* B-PAS should be used when performing health assessment in real-life patient encounters. They therefore felt prepared for and confident in using B-PAS to perform health assessment, as a part of fundamental care in nursing. This concurs with other research highlighting the benefits of multimedia learning material and virtual patients for learning health assessment, thereby increasing students’ preparedness for direct patient care [16, 17, 23, 27]. Despite the limitation of learning fundamental care and clinical skills in a technology-enhanced environment, the value of the study results can inspire the development of hybrid solutions for future clinical course designs in nursing education.

One of the study findings draws attention to the benefits and feasibility of the varying the different learning activities in a technology-enhanced learning environment. The combination of “traditional” academic assignments and technology-enhanced learning activities can be considered a strength of the RCC, according to the students and the faculty members. This concurs with other research highlighting that technology-enhanced learning material can be utilized in many ways in nursing education, to support learning fundamental care, health assessment, clinical skills, and preparedness for clinical practice [7, 14, 15, 20, 23]. Nevertheless, as Àlvarez-Nieto et al. [27] argue, it is important to invite both students and faculty members to critically evaluate the multimedia learning material created for educational purposes. This entails that students and faculty assess the relevance, design, format, and content quality of the learning material. The content of the suite of mLearning tools used in this study was co-designed with nursing students, also aiming to evaluate format, design, and quality [5]. The current study supports the relevance and usefulness of this Suite of mLearning tools: the students valued the virtual patient encounters, but also the MOOC with video-recorded examples of health assessment in encounters with patients. The faculty also valued the usefulness of the pedagogical approach in the RCC.

### Facilitation in virtual patient encounters should uncover the “red thread” within nursing education, fostering early development of clinical reasoning skills

**Fig. 5:**
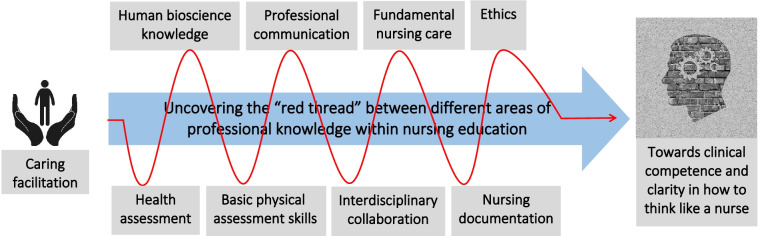
Uncovering the “red thread” within nursing education

The common link, here understood as the “red thread”—between professional knowledge, clinical skills, and clinical reasoning skills—is not always apparent for novice students. In this study, students and faculty members shared the opinion that the collaboration between the experienced facilitator, faculty members, and the students stimulated in-depth reflections and higher cognitive thinking, which made this “red thread” more evident. Six main characteristics were extracted from the qualitative data, highlighting central aspects of the facilitator role in the virtual patient encounters essential for student learning. The first five are: 1) explaining the rules of engagement; 2) clarifying different roles in the virtual simulation; 3) acknowledging students’ vulnerability; 4) communicating with care when correcting students’ incorrect answers; and 5) adjusting and modeling clinical reasoning skills. These aspects are supported by Deschênes et al. [20], who argue that pedagogical feedback and more Socratic ways of exploring one’s thinking and knowledge are closely related to the early phases of developing clinical reasoning skills. In addition, Gordon [28] emphasizes that debriefing (understood here as providing feedback) in the virtual simulation should aim to stimulate critical thinking and the connecting of the virtual patients’ clinical situations with real-world situations. The stop and pause action in the virtual simulation software offered this kind of exploration and linking of the virtual situation to real patient situations. This in turn linked different areas of professional knowledge, clinical skills, and clinical reasoning, which were then more consciously integrated into the students’ language, cognitive thinking, and learning processes. This brings forward the sixth central aspect of the facilitator role: 6) careful navigation of the in-depth exploration that uncovers the “red thread” between fundamental care and different areas of professional knowledge in nursing (Fig. 5). This explorative nature of the engagement with the virtual patients appealed to both students and faculty members in this study.

In this way, the students’ confidence can be appropriately stimulated in what was viewed by the students as a vulnerable learning situation. Deschênes et al. [20] support this pedagogical strategy and emphasize that when students put their thoughts and cognitive reasoning into words, it helps them integrate different areas of knowledge and develop clinical reasoning skills.

It was clear in both data sets that the students found that the involvement of faculty members in the simulation sessions added value to the total learning experience. This is also in line with Deschênes et al.’s [20] review showing that modeling clinical reasoning skills is highly beneficial for students learning these cognitive processes. The nursing students in current study also expressed that they would prefer to spend more time with the virtual patient and re-visit the clinical situation. This may boost their learning from earlier virtual patient encounters [16, 17].

### New technology-enhanced learning activities call for new pedagogical competence among faculty members

The COVID-19 lockdown and its consequences for the education sector were challenging for both nursing students and faculty members. In the qualitative interviews, the faculty members expressed the need to increase their confidence and competence regarding working in and using a technology-enhanced learning environment, such as the RCC. As Koehler and Mishra [4] underline, understanding the affordances of the chosen technology and its potential pedagogical advantages represent an important competency that faculty members need to acquire [4]. This also becomes visible when new technology-enhanced learning resources and educational innovations (e.g., virtual patients) are rapidly implemented without a firm grounding in pedagogical thinking on how best to utilize these to benefit teaching and learning [22]. The faculty members supervising the students in the current study did not have the opportunity to explore the technology-enhanced learning activities in advance of the RCC, due to the suddenness of the COVID-19 lockdown. As such, they lacked knowledge on how to utilize the pedagogical potential of these activities (e.g., the virtual patient encounters) to the fullest.

The faculty members also expressed a desire to develop this competence further, highlighting a need for the development of “train-the-trainer” course in this area. This draws attention to the complexity of the competencies needed to take advantage of the interaction between technology and pedagogy—as highlighted in the TPACK framework.

### Limitations and strengths

An important limitation of this study is that most of the first-year students lacked prior experience from clinical practice with which they could compare their perceived learning experiences from the RCC. However, the students attended learning activities in the skill lab on campus prior to the pandemic, where they worked with fellow students, training in fundamental care activities like B-PAS, and many other related clinical skills. Further, the students and faculty members provided valuable insight into learning experiences in the RCC and related to the different learning activities; these also highlight initial lessons learned to clarify pedagogical strategies that may be beneficial in disruptive times, with implications for post-pandemic educational practices. In addition, the study’s explorative convergent mixed-methods design strengthened the exploration of a new educational design. Since this was the first time a clinical course was redesigned into a technology-enhanced learning environment, the study had an explorative nature and the sample size was small. However, the participants had large information power, which justifies lower sample sizes [29]. This is important to highlight, as future research should investigate the effect of the different learning activities used in the RCC on student learning in a larger educational context. It would also be beneficial for future research to focus on comparing results from studies with students that were not impacted by the COVID-19 pandemic and the results from this study. Furthermore, the questionnaire used was designed to target the aims of this study and should be comprehensively validated in studies to come. A further limitation of the study is the lack of representation of both genders in the participant groups, as only one man was among the study participants.

## Conclusion

This study shows that in an RCC, a combination of traditional academic assignments, a Suite of mLearning tools, and virtual patients can help nursing students learn fundamental nursing care and be prepared for future clinical hours. The virtual patients played a significant role in providing learning situations that promote systematic health assessment to stimulate early development of clinical reasoning skills. Learning and engaging with virtual patients in a safe, virtual space appeared to have fostered students’ confidence, and supported their growth into their future professional role as a nurse. Further, the facilitator’s acknowledgment of students’ vulnerability and their caring facilitation in the virtual simulation played an important role in role modeling, and exploring coherence in professional knowledge, clinical skills and clinical reasoning skills. This study also highlights the gap in pedagogical competence among faculty members regarding facilitating learning in a technology-enhanced learning environment. The implications of this study should be considered in the context of post-pandemic times, when problems with sufficient access to “real-life clinical rotation activities” are mitigated. Nevertheless, there is a need to develop technological solutions and hybrid pedagogical designs that enhance students’ preparedness for patient encounters, which in turn will ensure patients integrity and safety.

## Supplementary Information

**Additional file 1.** God Reporting of A Mixed Methods Study (GRAMMS) guideline.

## Data Availability

The metadata from the qualitative and quantitative datasets that are used and analysed during the current study are available from the corresponding author on request.

## Abbreviations

mLearning: Mobile learning
TPACK: Technological, Pedagogical, and Content Knowledge framework
RCC: Redesigned clinical course
FoC: Fundamentals of Care framework
B-PAS: Basic physical assessment skills
CVCS: Commercial video conferencing system
ABCDE: Airways, Breathing, Circulation, Disability, and Exposure
MOOC: Massive open online course
LMS: Learning management system
