# Supplementary material for: The value of a redesigned clinical course during COVID-19 pandemic: an explorative convergent mixed-methods study
Source: BMC Nurs. 2022 Apr 24;21:94. doi: 10.1186/s12912-022-00872-8 (PMC9034970; doi:10.1186/s12912-022-00872-8)
Supplement: Supplementary file 1 — Additional file 1. God Reporting of A Mixed Methods Study (GRAMMS) guideline. [file 12912_2022_872_MOESM1_ESM.docx]

God Reporting of A Mixed Methods Study (GRAMMS) guideline

| **Guideline** | **Section: page** |
| --- | --- |
| Describe the justification for using a mixed methods approach to the research question | Method: under design p. 9 |
| Describe the design in terms of the purpose, priority and sequence of methods | Method: under design p. 9 and in figure 1 |
| Describe each method in terms of sampling, data collection and analysis | Method: p. 12, 13, 14, 15 and 16 |
| Describe where integration has occurred, how it has occurred and who has participated in it | Method: p. 15  Results: p. 17-26 |
| Describe any limitation of one method associated with the present of the other method | Results: p. 18 |
| Describe any insights gained from mixing or integrating methods | Strengths and limitation: p. 31 |

O'Cathain A, Murphy E, Nicholl J. (2008). The quality of mixed methods studies in health services research. J Health Serv Res Policy13: 92-98.
